# Quarterly Summary

**Published:** 1856-01

**Authors:** 


### QUARTERLY SUMMARY.

1.—*The Dental News Letter, for July,* 1855, contains the report of a case of treatment of the nerve of a tooth, and almost complete formation of a new crown. By A. B. Williams.

The tooth presented the appearance of a blackened mass of decayed bone, with three small points of sound teeth above the gum, on the anterior approximal, posterior part of the lingual, and on the posterior proximal surfaces. The spaces between these points extending below the gum. It was determined to excavate as much of the decayed bone as the painful nature of the operation would allow, and finally to remove the nerve; as the excavation of the diseased portion continued, it was discovered that the bone was *thoroughly* diseased, and the process of excavation was continued until only a thin lamina of bone covered the pulp, through which the operator thought the arsenic would readily act. The usual preparation of arsenic, sulphate of morphia, and creosote, covered with a bit of cotton dipped in alcoholic solution of gum sandarach. The pain was so great that at the end of six hours, the preparation was removed, affording, as the operator had always observed in similar cases of destruction and removal of the nerve, instantaneous relief. On the fifth day the character of the pain indicated a high grade of inflammation of the pulp; the bone covering it was removed, when the escape of a small quantity of purulent matter gave relief. Not until the twenty-first day was the pulp in proper condition to be removed, and five days after the removal of the nerve, it was replaced with gold. In the formation of the new crown, the operator's first step was to begin packing the gold at the anterior part of the buccal edge, and carry it over to the posterior part, at the same time extending it inward to the previously filled pulp cavity. The gold was built up in this way above the level of the gum, on that side, and then in the same manner upon the lingual side. This part of the operation over, the gold was packed uniformly throughout the cavity; the filling was completed and the operator having seen it since, has every reason to believe in the perfect success of the case.

---

2.—*Treatment of Alveolar Abscess.*—Prof. White reports in Dental News Letter the case of Master G.——, 14 years of age, and of good constitution, who applied, suffering from abscess of both the superior front incisor teeth, in which the nerves had been destroyed by a blow, and which had the median angles broken off, almost exposing the nerve cavities. The teeth were loose and discharging pus at several points between the gums and teeth. The gums were lanced opposite the roots at two points, and deep enough to meet the pus sacks, into which were inserted teats of cottons to effect a drainage of the pus in another direction, so that the gums might contract and grow to the necks of the teeth again. "In a few days ceased discharging around the necks of the teeth and escaped through the openings made in the gum. The gums grew firmer to the teeth, the swelling subsided, and in a few weeks the teats were left out, and renewed every other day. When the gums had healed, the pulp cavities were both opened from the fractured surfaces, which showed some signs of decay; the internal walls of the cavities were also considerably decayed; they were thoroughly cleansed and plugged to the apexes of the fangs with gold. They have not given the slightest pain or sign of abscess up to this time. The gums present a slightly blue appearance, but the teeth are as firm in their sockets as any in the mouth."—*Dental News Letter, July* 1, 1855.

---

### 3.—*Management of Light in the Performauce of Dental Operations.* By H. H. McQuillan.

The writer of this article points out the importance of the proper management of light to the dentist, and shows how a perfect knowledge of the anatomy and physiology of the eye, and of the laws governing light, and of the morbid changes induced by injudicious use of the organ, will enable us to guard against the injurious results alluded to, and after referring readers who are desirous of a more intimate knowledge of the subject to the works of Newton, Brewster and others, says,

"To the dentist a *clear* and *steady* light is a great desideratum. This can only be obtained with any certainty from the *northern sky.* The artist invariably makes choice of this light for his studio: this is true also of the daguerreotypist; and of the silk merchant desirous of displaying his goods to advantage. But it is of still greater importance to us when taking into consideration, that while it is reliable from its steadiness, it is also on account of its being a *reflected* light, less likely to prove injurious to the eye. A certain portion of the solar rays are *absorbed* by the northern sky, whilst those that are *reflected* constitute a clear and steady light, which, if properly managed, will not prove exacting upon the organ.

"That the southern light is too powerful, will be admitted by every operator of experience. I have found that even those who have contended that it is the best light to work in, invariably use ground glass windows to modify its power at certain periods of the day. Added to this, its vascillating character must act injuriously—every cloud that passes before the sun producing a marked change in the light. At one moment the operator may have a light of the greatest strength, and in a second, by the passage of a dark cloud, the object before him is only seen indistinctly, until the iris by enlarging the pupil accommodates the eye to the change; perhaps by the time this is effected the light again assumes its intensity, and the iris now, by a responsive action, decreases the size of the pupil, so as to secure perfect vision."

Dr. McQuillan closes the article with further arguments to prove the importance and advantage to be derived from the choice of the northern light by the dentist, and the disadvantages of all others.—*Dental News Letter, July,* 1855.

4. *Dental Hygiene,* a thecrematic paper. By Joseph E. Garretson.—The proposition supported in this article is, "That in connection with local cleanliness, a daily ablution of the ears and surrounding parts in water, the temperature of the season, will—the structure and material being fair, preserve, and to a degree arrest the decay of the teeth."—*News Letter, October,* 1855.

5. *On the Use of Amalgam for Filling Teeth.* By Elisha Townsend.—The author says, "intending to make no excuse for my own tardiness in recanting a professional error, lest I should be betrayed into a justification of it, and inviting my brethren in the same category with myself to come up to the mark with as little reserve, I propose to give my present views upon the much vexed question of the propriety of the use of amalgams for filling teeth." He then goes on to relate the cases of two teeth filled in the year 1834, with the amalgam of mercury and silver; these, with a few more, (intended as experiments,) included all filled with amalgam by Dr. T., up to September, 1854. The two cases reported were still good up to the last date. He says, that his attention was called to a reconsideration by a professional friend, whose experiments had proven the fallacy of the objections urged against it. The objections were, "1st, That it became black, and discolored the teeth. 2d, That it had produced salivation. 3d, That it contracted in hardening, and, therefore, did not fill the cavity, allowing moisture to surround it, and reproduce decay." "The first of these objections," he says, "*did* exist, but is entirely removed by the present method of preparing it, which consists in adding a large portion of pure tin, and then washing the compound thoroughly with absolute alcohol. The second objection, though answered with as much certainty, may not be so easy of conclusive proof.

"I have not been able to find any one, on whose judgment I could rely, and who really knew what ptyalism was, say he had met with a case of sure and marked character. Some had met with great tumefaction of the gums, looseness of the teeth, ulceration, &c., but we know that all these conditions are present in cases where no mercury has been employed. Stopping a carious tooth with a pledget of cotton, where there is disposition to alveolar abscess, will produce great swelling. Carelessness and want of cleanliness will allow accumulations of tartar, and a consequent loosening of the teeth, accompanied by a fetor of the breath, equal in disagreeableness to the odor of ptyalism, and not very readily distinguishable from it in all cases.

"I knew of one case which was reported as one of decided salivation, and confirmed also by two physicians, which was said to come from four large amalgam fillings; the mouth was very filthy, and no care had been taken by the patient to cleanse it. She was told it was so much diseased that she must lose all her teeth, perhaps her life. This filthy mouth was cleaned, the gums properly treated, and entirely restored to health, without even removing the fillings on whose devoted heads the anathema had been poured. The mercury of the amalgam was, therefore, clearly not answerable for the symptoms in the case, and I have not been able to find any other that would better warrant the charge against the material, at least no case or fact which requires us to rule it out of practice on this apprehension

"3d. It contracted in hardening, &c. This, by actual and careful experiment, it is proven not to do. It is well known that all substances or compounds which harden by the process of crystallization, rather expand than diminish their bulk. Now an amalgam of silver and mercury hardens by this process, and, therefore, cannot contract. That it does not contract is well proved, besides, by all experience of its use in dental cavities." After some further arguments in favor of the use of amalgam, the author gives Dr. Wm. Hunter's recipe for the preparation of amalgam, as follows: "4 parts of pure silver; 5 parts pure tin. The silver to be melted in a crucible, and when partially cooled, the melted slowly added, carefully shaking the crucible while pouring in the tin; a black flux is then thrown in, and the whole is reheated; then poured into the ingot. It should be filed with a sharp keen file, which is kept for the purpose, and used for no other. A good magnet should then be carefully passed through it to remove any portions of steel that may have separated from the file. It is then to be bottled and ready for use."—*Dental News Letter, October,* 1855.

---

6.—*Springing of Plates.* By H. S. Chase, M. D.—The author of this communication offers the following method for preventing springing after the teeth are fitted to the plate; he takes a strip of copper, one inch and a quarter in width, and No. 23 of the gauge in thickness, which is to be wrapped around the case, the copper to come within an eighth of an inch of the teeth; the ends are to be fastened by means of iron, copper or platina wire passed through holes in each end of the copper and twisted. Small shaped pieces are then to be cut out of the free edge all around, forming a kind of fringe which is to be bent at right angles, making a sort of cap just deep enough for the case. Now, after the inside of the cap is oiled, the case is adjusted and plaster and sand mixed very thin are poured between the teeth and sides of the cap. The wax may in a few minutes be removed, the teeth backed, and then five minutes is sufficient to dry for soldering. The small amount of plaster renders the soldering but the work of a moment.—*Dental News Letter, October,* 1855.

---

7.—*Management of Light.*—Dr. McQuillan, in the News Letter for October, 1855, offers some further remarks in continuation of this subject in the July number, he says,

"When operating upon cavities situated in the palatine surface of superior incisors and canines, and the posterior approximal surfaces of molars or bicuspids, either in the upper or lower jaw, every operator has no doubt frequently found some difficulty in getting sufficient light on the surface demanding attention, to obtain a perfect view of the cavity, owing to the shadow cast by the tooth operated on. The *stronger* the light, the more decided the *shadow.* To obviate this, when operating on the upper front teeth, I have found a small piece of white muslin, folded so as to cover the tongue, but not protruded from the mouth, of decided service. The light striking upon the muslin, is reflected to the palatine surface of the teeth, and defines the margins of the cavity perfectly. If the cavity is situated in the posterior approximal surface of a molar or bicuspid, a still smaller piece of muslin, held in place by the fore-finger back of the tooth operated on, will reflect sufficient light to give a perfect view of the cavity.

To prevent abrasion of the cuticle at the corners of the mouth, it was my habit for years, as it has been that of many operators, to protect the skin, by napkins, from coming in contact with the shaft of the instrument. For the last three years, however, I have discontinued the practice, from a conviction that the maintenance of such a course would be subjecting my eyes to a most injurious influence.

Owing to the napkin being white, none of the rays of light that fall on it are *absorbed*, but all are *reflected* directly into the eye of the operator."

8.—*Case of Chorea cured by the extraction of eight stumps.*—The Dental News Letter for October, 1855, contains the report of a case of Chorea cured by the extraction of eight stumps by Dr. Billard. The author says, "after an examination of the case which was one of what is commonly called St. Vitus' dance, that he "found several stumps in both jaws, the gums entirely covering some of them, and on pressure of the same it caused her great pain, and pus exuding upon the slightest pressure.* * * I proceeded to give ether, and it took a double quantity to make her insensible to pain. I then took out eight stumps, and some small pieces of dead alveoli, which had caused a continuous irritation of the parts." Since that time the author states that the paroxysms grew less frequent, and now the patient, Miss L., enjoys her usual health.

9.—Dental News Letter, for October, 1855, contains the following, which we clip entire:

"The patient was a boy, in whose left cheek the phagedenic ulceration commenced during recovery after scarlet fever. The internal use of chlorate of potassa was first tried, and persisted with, in ten grain doses, for several days, the disease meanwhile being unchecked. A single free application of the concentrated acid was then made to the part, and with the effect of completely arresting the morbid action. The induration of the surrounding part has since gradually subsided, and the sore is now almost healed. The case, althought not one of the most acute class, was yet of a character sufficiently alarming.—*Medical Times and Gazette.*

10.—*Anodyne Cement for Rendering Teeth Insensible to Pain.*—Mr. J. P. Clark, of London, with the object of rendering diseased teeth insensible to pain, previous to the operation of stopping them permanently with metal, or filing away the carious parts, recommends the application of a paste, composed of Canada balsam and slacked lime, which is to be inserted into the hollow tooth like a pill. Mr. Clark states, that this preparation affords immediate relief in all but chronic cases of inflammation. He says:

"If fresh pills be inserted as often as the old ones wear out, or are removed on the return of pain, in order, like an abscess, to allow the escape of matter or blood, and this practice continued, the teeth will become as insensible to touch as the soundest, and may then be permanently stopped, and otherwise treated in the usual way without the infliction of pain."—*Dublin Hospital Gazette.*

### CHEMICAL.

11.—*Adipocire—Its Formation.*—Dr. Charles M. Wetherill read last winter an able memoir on this subject before the American Philosophical Society, which has been published in the Society's Transactions. The author has performed a number of experiments tending to determine the manner in which adipocire is formed and the materials which go to constitute it. The general belief among chemists since this substance was first carefully examined by Fourcroy, has been that it is formed out of all animal substances except hair, nails and bones. Lord Bacon affirms in one of his works, that nearly all flesh may be turned into a fatty substance by cutting it into pieces and putting it into a glass covered with parchment, then letting the glass stand six or seven hours in boiling water. In 1786-7 the Cemetiere des Innocens, at Paris, having been opened for the removal of the bodies to another grave yard,

Fourcroy had ample means of observing the circumstances under which adipocire is formed. He found an abundance of the substance, especially in the ditches where the slightly made coffins of the poorer classes had been piled one upon another; the trench being open for some time till it was filled with bodies when it was slightly covered with earth. On opening the trenches after some fifteen years the bodies were converted into adipocire; they were flattened by mutual pressure and had impressions on their surface of the grave clothes. George Smith Gibbes, a few years afterwards, observed that in Oxford, in the pits were thrown the remains of dissections, and at the bottom of which flowed a gentle current of water, large quantities of adipocire were formed; and placing a piece of beef in the river in a box pierced with holes, this substance also resulted. He states that nitric acid will affect the same changes in a few days. Bostock seemed to prove by some experiments which he instituted, that muscular fibre could be converted into fat by the action of nitric acid. Chevreul, on repeating his experiments, could not obtain fat from pure fibrine; but Von Bibra, as the result of many experiments, concluded that muscle, under certain circumstances, is changed to fat, and Blondeau arrived at the same conclusion from an examination of the cheese manufactured at Roquefort. The cheese when first made, contained 1,200 of its weight of fat, but after two months in dark, cool, damp cellars, the caseine was almost wholly converted into fat. A piece of beef, after two months in the cellar, surrounded with paste and slightly salted, was found changed, for the greater part, into a fatty body, presenting the greatest analogy to hog's-lard.

The question is an interesting one in a physiological point of view. Some experiments recently performed by Liebig, Bopp, Keller and others, rendered possible the formation of fat from albumen, fibrin and caseine. It is certain that herbivorous animals possess more fat than is taken in their food, but it has generally been conceded that the excess comes from the sugar and other non-nitrogenized compounds. In like manner where organs in the animal body have undergone the fatty degeneration, the oil is supposed to be derived from other parts of the system, rather than formed in the part by a metamorphosis of the nitrogenous compounds.

Dr. Wetherill engaged in the study of adipocire with reference to this question. In his investigations he found that this substance seemed to be formed under opposite circumstances. In some graves it was discovered, and in others contiguous to them decomposition had advanced to its full extent, leaving nothing but the skeleton. The preservation of some bodies seems, indeed, inexplicable in the present state of our knowledge, as, for example, the case of General Washington, who, having reposed in his tomb for more than forty years, was so perfectly preserved as to have been recognized from the resemblance of his portraits. When Dr. Wetherill engaged in the examination of adipocire he was inclined to the belief that it was a result of the blood-forming substances, but his experiments have brought him to a different conclusion, namely, that the higher members of the series of fatty acids do not result from the putrefaction of proteine compounds; and as corroborative of this view he mentions, that in all cases where adipocire has been found, the corpse was of a large and fat person. In grave-yards, he has remarked, if the proportion of flesh to fat be large, and especially if the nature of the ground be such as to prevent the escape of the decomposed matter, as by draining, no adipocire is formed, but the fat undergoes full decomposition. The microscope sustains his experiments, inasmuch as it fails to trace any arrangement of the fatty particles into fibres or rows, such as would here and there be seen if the fat came from muscles. The original fat of the body, Dr. Wetherell concludes, according to circumstances at burial, either partakes of a decomposition by which its elements escape, or else, losing its glycerine and most of its oleic acid, becomes gradually converted into adipocire. We do not suppose that his experiments settle the question, but to our mind they are nearly conclusive.
*Western Journal.*

---

12.—*Ozone in Epidemics.*—[Translated from Review de Thérapeutique Medico-Chirurgicale, for the New Orleans Med. and Sur. Journal, by Mrs. M. E. V.]

M. Wolf, director of the observatory at Berne, announces in a letter addressed to the Academy of Sciences that, according to his personal observation, a rapid diminution of ozone in the air is (if not always, at least usually) followed by a considerable augmentation of mortality.

What is ozone? This is the question.

We answer with pleasure, for a new subject is in question, which is worthy attention under several aspects, and which recommends itself equally to the chemist, meteorologist, and physician. Henceforth the study of the variations of this new principle enters into daily meteorological observations under the same claim as that of temperature, atmospheric pressure, etc.

Ozone is nothing else than oxygen itself; this gas which enters for twenty-one centiémes (·21) in the composition of the air we breathe. But is oxygen so different from that to which chemists are accustomed to give that name, that they have a great deal of trouble to recognise it under its disguise.

For instance, oxygen is without scent, as every one knows; ozone on the contrary has a strong odor. It is by its scent, indeed, that its presence became known to observers; its name being taken from the Greek, reminds you of this property. Its odor oftentimes partakes of those of chlorine, of phosphorus, and of burning sulphur mixed with air. It manifests itself in the discharges attendant on the turning the plate of an electrical machine, or when there is a peal of thunder.

But the new and permanent qualities which oxygen acquires in becoming ozone are not confined to this; we know, for instance, that ordinary oxygen only combines slowly with mercury at the usual temperature; ozone, on the contrary, combines rapidly with this metal. In a word, the oxydizing qualities of ozone are much more energetic than those of ordinary oxygen.

Van Marum was the first who found this remarkable substance. It was in 1785; having at his disposal the great apparatus belonging to the museum of Teyler, he excited sparks in a tube full of oxygen. A quarter of an hour afterwards, after 5,000 sparks, the oxygen had acquired a strong scent which he regarded as being very clearly that of electricity.

From 1785 to 1840, these remarkable experiments were completely lost sight of. In the course of the last year, M. de Schoenbein, professor of chemistry at Bale and inventor of the gun-cotton, decomposing water by means of an electrical battery, remarked that the production of hydrogen gas was accompanied by a very peculiar odor. He published a memoir on the subject. What was this new substance? Was it a simple element—was it an oxygenated product of azote or of hydrogen? The ingenious chemist left the question undecided, but he gave this odorous substance the name of *ozone.*

In 1851, two *savants* of Geneva, MM. Marignac and De la Rive, concluded after a series of experiments that ozone is nothing but oxygen in the peculiar state of chemical activity given to it by electricity. Berzelius and M. Faraday believed equally that there is here, an isomeric or allotropic state, that is to say, a simple modification of the oxygen. Finally, in the same year, 1851, M. Schoenbein, who, on discussing this interesting question for the third time, agreed with MM. Marignac and La Rive.

Nevertheless, the majority of chemists admitted it hesitatingly. New experiments were necessary. Those made by MM. E. Frémy and Edmond Becquerel, in 1852, seemed to remove all doubts. They have shown, in confirmation of the experiments before mentioned, that electricity, acting on oxygen, develops new qualities in it; and these gentlemen have proposed changing the name of ozone into that of electrified oxygen (*Oyzéne électrisé.*)

Ozone then is nothing but a peculiar form of oxygen. Thus we are shown the changes that simple and compound substances experience in their most essential qualities from the force of exterior circumstances. The changes in oxygen that electricity causes in this case are comparable, in fact, to those produced by the solar rays on chlorine, whose affinities become much more energetic, and to those which heat develops in sulphur, phosphorus and carbon, whose colors, consistence, solubility and affinities they modify, and in many compound metallic oxyds, for instance, experience under its influence isomeric transformations. Thus, this collection of mysterious facts is of powerful interest and fully studied, will doubtlessly modify the classic ideas already much shaken as to the pretended number of simple substances which are thus found numerous by combination.

Once admit that by the agency of electric sparks oxygen can enter into a state of chemical activity, then we must ask, if this change which we produce in our laboratories, is not produced spontaneously in the air; the more so as we cannot doubt that the atmosphere incessantly agitated by storms, experiences these chemical changes we speak of. This is a matter, however, of which one does not soon acquire a certainty.

Since 1850, M. Schoenbein has proved that ozone decomposes the iodide of potassium, and he proved that a band of starched paper containing a small quantity of this salt, constitutes the most powerful, delicate reagent for discovering the presence of this new substance. A strip of paper prepared in this manner and exposed to the air, soon revealed, in changing from white, which was its original color before the experiment, to a blue of more or less intensity, the presence of ozone. That ozone then exists naturally in the air, is a point demonstrated; but does it always exist in the same proportions? The contrary is evident. Of what importance is it then, to study its variations? To do this it was necessary to bring back the observations to a common mode of comparison; nothing proved more easy.

Observers first arranged an ozonometric scale by dividing into a certain number of parts or degrees, the chromatic space comprised between the white which answers to the absence of ozone and the most intense blue that ozone can produce, at its maximum on ozonoscopic paper, putting on simple iodine. Ten was the number of visions adopted, and, this done, they possessed an *ozonoscope* by means of which they could measure the daily variations of the atmospheric ozone, as they could by means of the thermometer and barometer measure the daily changes of the temperature, and atmospheric pressure.

Several philosophers, knowing the importance of this new branch of meteorological observation, have pursued it. MM. Bœckel at Strasburg, Simonin, Sen., at Nancy; Wolf, at Beane; Billard, at Corbigny; Schapter and Besluber, in Germany; Gailliard, in America, etc., are among these observers.

So that here is a substance, whose existence was not suspected a few years ago, and one which acts on us and on all animated nature. How is it possible to doubt as to the intensity of its action? How can one doubt but that considerable variations in the oxydizing power of respirable gas have a powerful influence on respiration, and, in consequence, on all the vital functions?

An American physician, M. E. S. Gailliard, finds a connection between ozone in atmospheric air and the appearance of intermittent fevers.

According to Dr. Bœckel, malaria always shows itself with the zero of the ozonoscope, and the same thing takes place when peludal fevers prevail.

According to M. Schoenbein, a considerable quantity of ozone was observed in the air of Berlin during the epidemic *Grippe* and during a medical constitution of the air predisposing to affections of the chest; and the reverse took place under the prevalence of the gastric constitutiou.

According to the same observer, ozone was completely wanting in the atmosphere of the same city during a cholera epidemic.

According to M. Bœckel, the same thing occurred at Strasburg. The appearance of the cholera coincided with the absence of ozone, and the ozone reappeared as the cholera decreased.

M. Billiard regards the diminution of ozone as the first cause of this terrible malady.

Finally, without going so far, M. Wolf, in the letter which occasioned this article, confirms, as to the city of Berne in which he resides, the observations made at Strasburg by Dr. Bœckel.

It appears likely to us that ozone has not only great physiological parts to perform, but it is necessary to employ it in explaining several physical and chemical phenomena, such for instance, as the productions of nitric acid in the atmosphere, and the disengagement of the odor which so often accompanies thunder.

*(L'Ami des Sciences.)* VICTOR MEUNIER.

[Addendum, from the *Gaz. Hebdon. de Med.* of May 11, 1855.]

*Ozone and Cholera.*—Having procured the daily list of deaths from cholera at Aarau, in Switzerland, from the 15th of August to the 14th of October, 1854, M. Wolf, director of the observatory at Berne, has classed the days in which no deaths occured—those in which there were one or two, and finally those in which there were three or more: "I have found," says he, "that the corresponding mean of the actions of ozone at Berne is—

| | | |
|---|---|---|
| For the days of the | first class | 6.48; |
| " | second class | 5.48; |
| " | third class | 4.58; |

"I conclude from this," says M. Wolf, "that the cholera, to say the least, is very much favored by the diminution of ozone."—*L'Ami des Sciences.*

